# Multisensory visuo-tactile context learning enhances the guidance of unisensory visual search

**DOI:** 10.1038/s41598-021-88946-6

**Published:** 2021-05-03

**Authors:** Siyi Chen, Zhuanghua Shi, Hermann J. Müller, Thomas Geyer

**Affiliations:** Allgemeine Und Experimentelle Psychologie, Department Psychologie, Ludwig-Maximilians-Universität München, Leopoldstr. 13, 80802 München, Germany

**Keywords:** Human behaviour, Computational biology and bioinformatics, Environmental sciences

## Abstract

Does multisensory distractor-target context learning enhance visual search over and above unisensory learning? To address this, we had participants perform a visual search task under both uni- and multisensory conditions. Search arrays consisted of one Gabor target that differed from three homogeneous distractors in orientation; participants had to discriminate the target’s orientation. In the multisensory session, additional tactile (vibration-pattern) stimulation was delivered to two fingers of each hand, with the odd-one-out tactile target and the distractors co-located with the corresponding visual items in half the trials; the other half presented the visual array only. In both sessions, the visual target was embedded within identical (repeated) spatial arrangements of distractors in half of the trials. The results revealed faster response times to targets in repeated versus non-repeated arrays, evidencing ‘contextual cueing’. This effect was enhanced in the multisensory session—importantly, even when the visual arrays presented without concurrent tactile stimulation. Drift–diffusion modeling confirmed that contextual cueing increased the rate at which task-relevant information was accumulated, as well as decreasing the amount of evidence required for a response decision. Importantly, multisensory learning selectively enhanced the evidence-accumulation rate, expediting target detection even when the context memories were triggered by visual stimuli alone.

## Introduction

For finding a searched-for target object within a recurrent environment, a potentially useful ‘strategy’ of the visual system would be to learn the critical target together with the non-target, or ‘distractor’, context in which it is consistently embedded. Indeed, contextual learning has been shown to facilitate visual processing when a learnt target-distractor pattern re-occurs on later occasions. For instance, Chun and Jiang^1^, in their seminal study, presented participants with search arrays containing a target letter “T” amongst a set of distractor letters “L”. Critically, in half of the trials, the spatial arrangements of the distractor and target stimuli were repeated (i.e., ‘old’ contexts), whereas in the other half, the distractor locations were generated anew on each trial (i.e., non-repeated, ‘new’ contexts). They observed that visual search was facilitated for old, as compared to new, contexts—an effect termed “contextual cueing”. Just a few (some 3–6) repetitions of a target presented at a fixed location relative to an invariant distractor arrangement suffice to produce a significant contextual-cueing effect, and the effect appeared to be ‘implicit’ (and ‘automatic’) in nature: explicit recognition of old configurations was not significantly above chance level. And attesting to the automaticity of contextual cueing, Zinchenko et al.^2^ recently showed that, once acquired, contextual cues continue to bias attention to the originally learnt target location, even after consistent re-positioning of the target to a new location. Moreover, contextual cueing turned out to be a highly reliable phenomenon (for replications, see, e.g.,^3–8^), likely rendering it suitable for studying the behavioral and computational mechanisms involved in statistical learning not only within but also across sensory modalities (e.g.,^9^).

**Figure 1:**
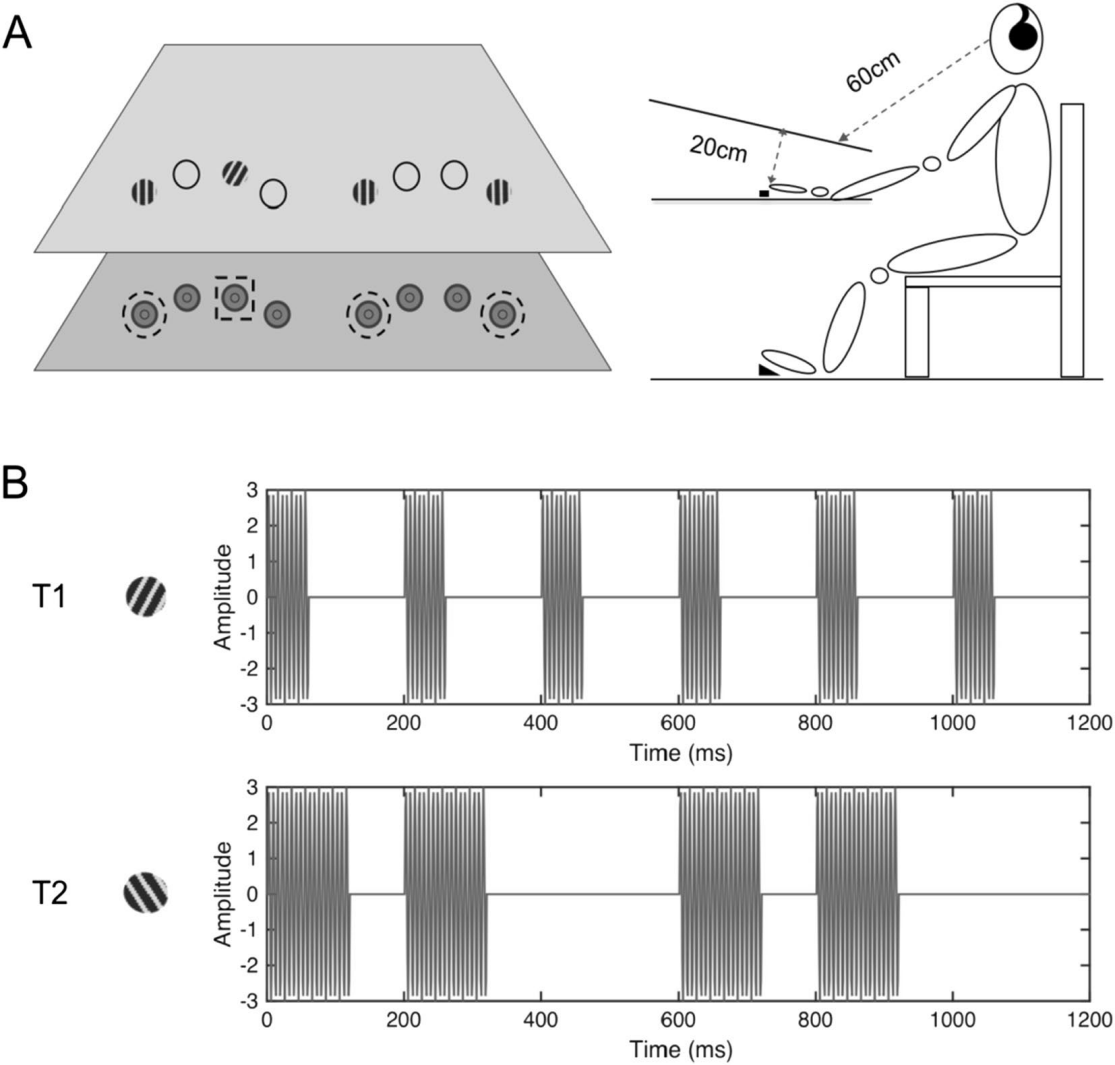
Illustration of the experimental setup and stimuli. (**A**) The visual and tactile stimuli were presented at spatially corresponding locations on an upper and lower presentation plane (from the first-order perspective of the participant). Each search item, whether target or distractor, was defined by a certain visual and tactile stimulation arising at the same (one-to-one mapped) location. The dashed square indicates the location of the singleton tactile target and dashed circles the locations of the tactile distractors. Visual stimuli were presented on a white canvas surface tilted about 20° towards the observer. The viewing distance was 60 cm. (**B**) depicts the identity of the two possible visual-tactile targets. The upper panel indicates the orientation and waveform of target T1: a right-tilted Gabor patch, and a 5-Hz tactile stimulus with a 30% duty cycle delivered via 150-Hz vibration. The lower panel illustrates target T2: a left-tilted Gabor patch, and a 5-Hz tactile target stimulus with 60% duty cycle composed of 150-Hz vibration. The distractors are vertical bars constantly vibrating at 150 Hz. The pairing of a given visual and tactile target was balanced across observers: half of observers had right- (left-)tilted visual targets in combination with (duty cycles of) 30% (60%) tactile targets, and vice versa for the other half.

Given the ultimate goal of contextual cueing research is to understand the guidance of attention during search through familiar, natural scenes, models of contextual cueing will need to be extended to reflect the fact that real-world search almost always occurs in multisensory environments (e.g.,^10^). Indeed, recent work has shown that contextual cueing is not confined to the visual modality. For instance, implementing a new multisensory search scenario, Chen and collaborators^11^ demonstrated context-based facilitation of search when the target was defined in the visual modality and the predictive (old) distractor arrangement in the tactile modality. In more detail, the predictive tactile distractors were presented at participants’ finger locations while they searched for a visual odd-one-out target in a co-located, homogeneous visual array (co-location means that, essentially, the visual and tactile items occupied the same spatial locations, though technically they were presented on slightly offset depth planes; see Fig. 1A). Of note, crossmodal, tactile-to-visual contextual cueing was most pronounced when the predictive tactile items preceded the visual target. To some extent, this is attributable to the fact that more time is required to single out and discriminate the frequency pattern of the tactile target (pattern defined in time) as compared to singling out and discriminating the visual target’s (stationary) Gabor orientation^12,13^. However, in addition to the generally slower processing of the touch-defined target and distractor stimuli, tests using hand-gesture manipulations, such as flipped hands, revealed that tactile-to-visual contextual cueing is mediated by an environmental reference frame. This suggests that part of the tactile lead time is required for the tactile item configuration to be remapped from an initially somatotopically sensed format^14^ into a common (visual) spatial representation for crossmodal contextual facilitation to occur^13,15^. Thus, there is positive affirmative evidence that contextual memories established in one modality (e.g., touch) can interact with search processes in another modality (e.g., vision).

However, in real-life scenarios, contextual cues would rarely be provided by a single, fixed sensory modality (such as the tactile modality in Chen et al.^11,13^). Rather, perceptual learning is more likely to involve distributed processing of information from multiple sensory modalities for regularities that eventually come to be stored in long-term (in our case: context) memory, and this may be the case even if only one modality is ultimately task- (i.e., response-) relevant. Accordingly, in the present study, we made the visual modality task-relevant and asked whether exposure to multisensory contexts that consist of repetitions (of the locations) of both visual and tactile item patterns would engender more robust contextual memories relative to unisensory contexts, specifically, memories deriving from repetitions of visuo-spatial arrangements alone.

That is, departing from the paradigm and findings of Chen et al.^11,13^, we hypothesized that presentation of context cues from both—the visual and tactile—modalities during learning would combine to improve contextual memory, over and above the level achieved with visual contexts alone. Moreover, these influences might be so strong that they become measurable in a subsequent unisensory ‘consolidation’ phase (following training with multisensory stimulation), when the visual target is encountered in a pure visual environment without concurrent tactile stimulation. Relating to this issue, it has been shown that multisensory exposure enables stimuli to be encoded in multisensory representations, thus activating a larger network of brain areas than those invoked by unisensory encoding^16^. For instance, in Zilber et al.^17^, a group of participants who received multisensory (audiovisual) training with congruent acoustic textures outperformed two other groups trained with either a visual-only task or a visual task with auditory noise, in showing superior post- versus pre-training performance in tests of the same visual discrimination task presented without sound. Neuronally, apart from the activated region common to all three groups, visual motion and multisensory cortical areas were additionally activated in the multisensory training group, with the activity in multisensory cortices correlating with post-training performance^17^. Thus, it is possible that both uni-and multisensory neural systems contribute to crossmodal effects (see also^18^). However, only little is known about how brain activations due to multisensory experience are mapped onto cognitive (including computational) processes, such as how to guide attention to task-relevant objects in our complex, multisensory environment and to select appropriate, goal-directed actions therein. Moreover, to our knowledge, no crossmodal study has ever addressed the issue of multisensory training on spatial context learning in a visual search task. Given that multisensory experience leads to activations in multiple sensory regions—and, importantly, this is seen even when the task-relevant stimuli are presented in isolation—we would expect visuo-spatial contextual memories, too, to be strengthened by multisensory stimulation.

Thus, in the current study, we examined the impact of multisensory (relative to unisensory) experiences on statistical context learning in a visual search task. Using the approach of Chen et al.^11^, we tested contextual cueing in two separate, uni- and multisensory, sessions. In the unisensory session, the search task was a visual contextual-cueing task, while in the multisensory session the unisensory visual and the multisensory visuo-tactile stimulus arrays were presented randomly intermixed across trials. Our dependent variables were the contextual-cueing effects (mean RT new displays minus mean RT old displays) obtained on multisensory and unisensory trials as well as the parameters from exponential and drift–diffusion modeling (DDM) of individual observers’ RT data from these trials.

Concerning the latter, Chun and Jiang^19^ have shown that the decrease in RTs attributable to contextual cueing can be described almost perfectly by an exponential (i.e., power) function, where the critical parameter indicative of learning is provided by the amplitude of the decrease. Thus, to assess the beneficial effect of multisensory over unisensory learning, we fitted the RT data from unisensory and multisensory trials with exponential functions. Moreover, manual RTs in visual search tasks typically reflect the end result of multiple—perceptual and response-related—processing stages ^20^. Some have argued that contextual cueing influences an ‘early’, target selection stage^21–23^: observers learn to associate the positions of distractor elements in repeated displays with the location of the target, which expedites the search stage of the task (in line with Chun and Jiang’s attentional-guidance account^1^). Others have advocated a response-facilitation account^24,25^, according to which the cueing benefits arise ‘late’ in processing, at the response selection stage, due to observers adopting a more relaxed decision threshold for repeated, as compared to novel, search displays. Yet, some others have suggested that cueing benefits occur at both early and late stages^2,26,27^. Importantly, going beyond these studies, by comparing estimated DDM parameters among different contexts (e.g., old vs. new, unisensory vs. multisensory), in the present study we aimed to dissociate the attentional-guidance and response-selection accounts of contextual cueing following multisensory (and unisensory) exposure.

## Method

### Participants

14 naive observers (24.9 ± 3.3 years; 7 males), all right-handed and all with normal or corrected-to-normal vision and normal tactile sensation, were recruited from the LMU Munich student population. Observers were paid nine euro per hour for their participation. They all provided written informed consent prior to the experiment. The study was approved by the Ethics Committee of the LMU Munich Faculty of Psychology and Pedagogics. The experiments were performed in accordance with relevant guidelines and regulations. The sample size was determined based on previous studies of statistical context learning in tactile and visual search tasks^6,11,28^, aiming for 85% power to detect a relatively large effect (f(U) = 0.8) in a repeated-measures analysis of variance (ANOVA; *η*_*p*_^2^ = 0.4) with an level of 0.05. Power estimates were computed using G*Power^29^.

### Stimuli

Visual stimuli consisted of four Gabor patches (Michelson contrast of 0.96, the spatial frequency of 2 cpd) and four empty circles, each subtending 1.8° of visual angle, presented on a grey background (36.4 cd/m^2^). Of the four Gabor patches, one patch was the visual target and the other three were homogeneous visual distractor patches. The orientation of the distractor Gabors was vertical, and the orientation of the target deviated ± 9.2° from the vertical^11^. The visual items were presented at eight ‘virtual’ finger locations on an upper plane (see Fig. 1A), with a distance of ca. 1.9° of visual angle between adjacent items. Vibrotactile stimulations were delivered at, relative to the visual items, spatially corresponding finger locations, with one finger being the tactile target and the other three tactile distractor fingers (see Fig. 1A). Participants placed their eight fingers (except the thumbs) on eight solenoid actuators (a diameter of 1.8 cm)^11,14,28^. The positions of the actuators were collocated to the visual stimuli from first perspective view of the participant, though they were individually fine-adjusted to fit participants’ hands (i.e., varying within ~ 0.5 cm in the Y- and X-directions relative to a distance of 2 cm between adjacent actuators), ensuring optimal comfort and performance. The vibrotactile distractors were constant 150-Hz vibrations, while the vibrotactile targets were defined by a different square-wave modulation (see Fig. 1B): target 1 (T1) was a 5-Hz square wave with 30% duty cycle, composed of 150-Hz vibrations; target 2 (T2) was a 5-Hz square wave with an average 60% duty cycle, also made up of 150-Hz vibrations. To make T2 distinguishable from T1, a blank gap of 200 ms was inserted between every two cycles in T2 (thus, the mean frequency of T2 was 3.3 Hz). The actuators activated lodged metal tips vibrating a pin by 2–3 mm upon the magnetization of the solenoid coils, controlled by a 10-Channel Tactor Amplifier (Dancer Design) connected to the computer with a MOTU analog-output card. Of note, the waveform type of the tactile target and the orientation type of the visual target were paired for each observer in the multisensory session and the combination was counterbalanced between participants (e.g., for half of the participants, T1 was coupled with a right-oriented visual Gabor item and T2 was associated with a left-oriented Gabor stimulus, and vice versa for the other half). Participants responded by pressing one of two foot pedals, in both the uni- and multisensory sessions. Moreover, in the multisensory session, the response to the visual and tactile targets was always consistent: for half of the participants a T1/ left Gabor required a left foot pedal press and a T2/ right Gabor required a right foot pedal press (and vice versa for the other half). Participants were asked to wear headphones (Philips SHL4000, 30-mm speaker drive) through which white noise (65 dBA) was delivered to mask the (otherwise audible) sound produced by the tactile vibration. In the multisensory session, the white noise started and stopped together with the vibrations; in the unisensory session, it was presented for the same duration on each trial as in the multisensory session.

### Procedure

#### Practice session

Following written and verbal instructions, participants were equipped with headphones and comfortably seated on a chair with their fingertips (except the thumbs) gently placed on the stimulators on the lower plane while looking at the visual display projected onto the upper plane (screen). Participants first learned the mapping between the foot (i.e., response) pedals and the targets. The target-pedal assignment was fixed for each participant but counterbalanced across participants. The practice session consisted of four tasks: (1) visual search; (2) tactile target identification; (3) tactile search; and (4) multisensory search. Each practice task consisted of 32 trials, apart from the last, multisensory search task, which had 64 trials (with half of trials containing visual targets and the other half redundantly defined, visual-tactile targets). Participants had to reach a response accuracy of 85% in a given task before proceeding to the next task.

**Figure 2:**
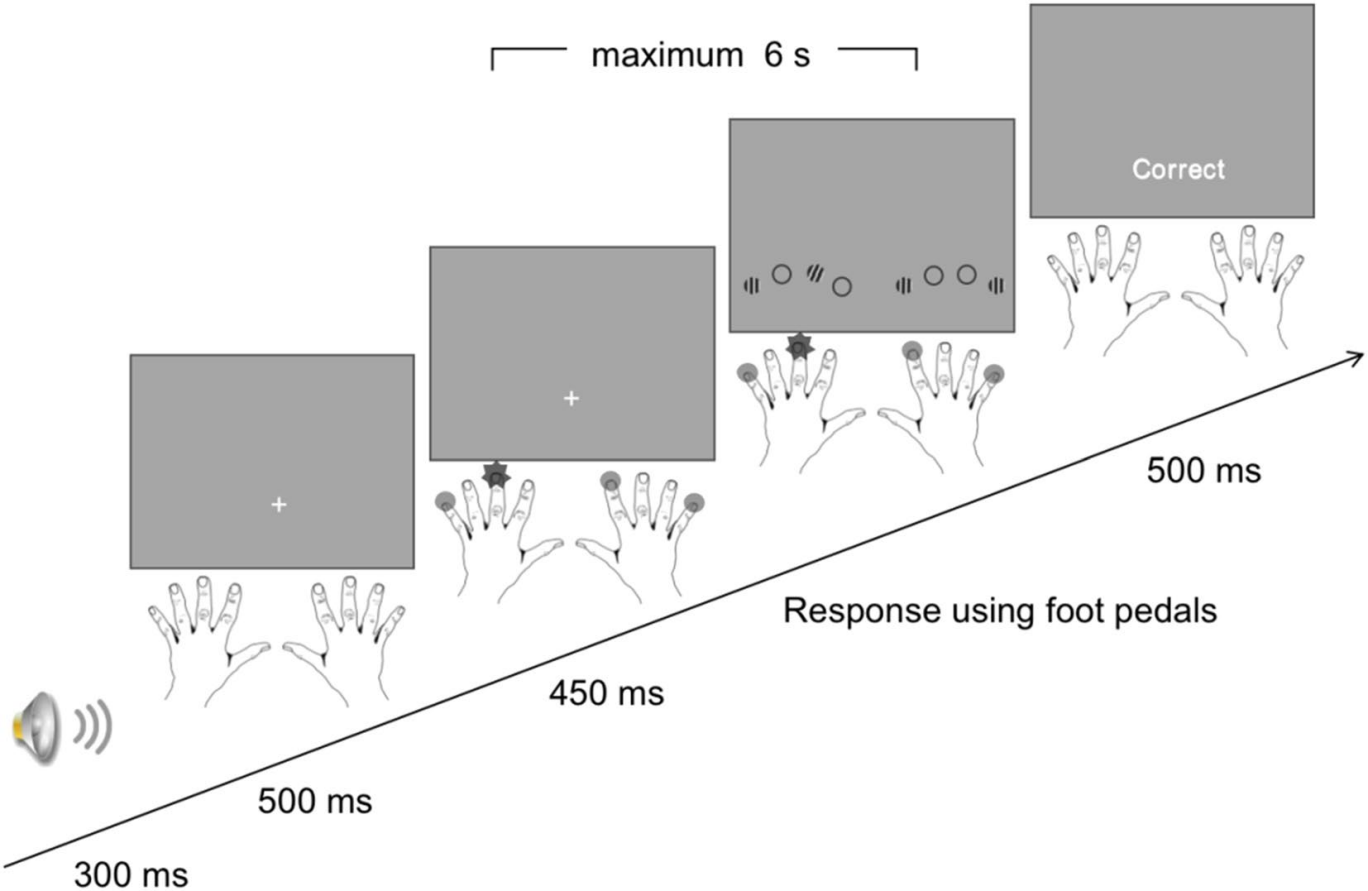
An example stimulus sequence of a visual-tactile search display from the multisensory session. After the initial auditory beep, tactile stimuli were presented for 450 ms prior to the onset of the visual items. The dark ‘star’ represents the tactile singleton (target) finger, and the light grey disks the non-singleton (distractor) fingers. The four visual items were Gabor patches presented at, relative to the fingers, corresponding locations. The visual target was the single left- vs. right-tilted Gabor patch, amongst the three vertical distractor Gabor patches. Observers’ task was to discriminate the orientation of the visual Gabor target by pressing the corresponding foot pedal. A feedback display (together with a warning beep in case of an erroneous response) was presented after the response. The maximum stimulus duration was 6 s. In the unisensory session, only visual Gabor items (and the rings) were presented.

In the visual search task, there were eight visual items (four Gabors and four ‘empty’ circles) presented on the screen. Participants were asked to identify the Gabor orientation (tilted to the left or the right) as rapidly as possible by pressing the corresponding foot pedal. In the tactile target identification task, one vibrotactile target (either T1 or T2) was delivered to one of the eight fingers randomly, and participants were asked to respond as quickly as possible by pressing the corresponding foot pedal. In addition, during the first block, participants received visual cues (the symbols “T1” or “T2” on the screen), helping them to associate the tactile target with its respective response pedal. In the following ‘tactile search’ task, the vibrotactile target was delivered together with three vibrotactile distractors in the tactile search array. Two fingers were stimulated on each hand. Participants had to identify T1 or T2 as quickly and as accurately as possible by pressing the associated foot pedal. In the final, multisensory search task, there were 50% visual trials and 50% visual-tactile trials, randomly interleaved. The visual trials were identical to the visual-search practice task: only visual items were presented to participants. The visual-tactile task had visual and tactile items (one target and three distractions from each sensory modality; see Fig. 1A). Tactile items were presented 450 ms prior to the visual stimuli (in order to compensate for processing differences across modalities and to allow tactile-to-visual remapping to take place^11,13^; see Fig. 2). This design aspect was based on our previous studys^13^ in which we manipulated the type of-crossed versus uncrossed –response-hand requirements in a tactile localization task (the former necessitating remapping in order to respond to the external—left vs. right—location of the tactile target) and found that tactile remapping took some 360 ms to be completed. Given that tactile localization requires some additional 140 ms compared to visual localization^13^, the combined estimate (of 500 ms) matches the current tactile preview time of 450 ms) quite well. Importantly, although the multisensory session had two targets defined in the two different sensory modalities, participants were expressly instructed to concentrate on the visual task. This instruction was meant to ensure that the visual task was near-identical across the uni- and multisensory trials, permitting us to examine for beneficial effects of multisensory versus visual stimulation on statistical context learning in a *single, visual modality.* The target-pedal assignment was counterbalanced across participants, while being consistent for the uni-and multisensory search tasks for a given participant.

#### Experimental session

Immediately following the practice session, each participant performed two experimental sessions: unisensory and multisensory. In the unisensory session, only unisensory-visual trials were presented; in the multisensory session, both unisensory-visual and multisensory-visuotactile trials. The trial procedure of the experimental tasks was identical to the respective practice tasks (see Fig. 2).

Each trial began with a 600-Hz beep (65 dBA) for 300 ms, followed by a short blank interval of 500 ms. A search display (either visual or visual-tactile) was then presented either until a response was made or a maximum display duration of 6000 ms had elapsed. For visual-tactile search displays, the tactile stimuli were presented 450 ms prior to the visual stimuli. Responses were recorded using foot pedals (Heijo Research Electronics, UK). Following observers’ manual responses, a feedback display with the words “correct” or “wrong” was presented in the center of the display for 500 ms. In addition, an error warning beep (2500 Hz, 85 dBA, 900 ms) was delivered simultaneously together with the visual feedback for incorrect responses or non-response time-out trials. After an inter-trial interval of 1000 to 1500 ms, the next trial began (see Fig. 2).

Participants were instructed to focus on the visual information. The reaction time (RT) was recorded from the onset of the visual stimuli. Half of the participants started with the unisensory session, the other half with the multisensory session. The unisensory session consisted of 128 trials for each target orientation (256 trials in total). The multisensory session consisted of 128 visual only trials (with 64 trials for each target orientation) and 128 visual-tactile trials (with 64 trials for each target orientation). Thus, participants received the same amount of visual stimulation in the two sessions (256 trials each). The experiment was divided into 32 blocks of 8 trials each, in each session. After every two blocks, double beeps (2 × 200 ms, 1000 Hz, 72 dBA, separated by an 800 ms silent interval) were sounded, upon which information about the mean accuracy attained in the previous two blocks was shown in the center of the screen for 1000 ms.

### Design

**Figure 3:**
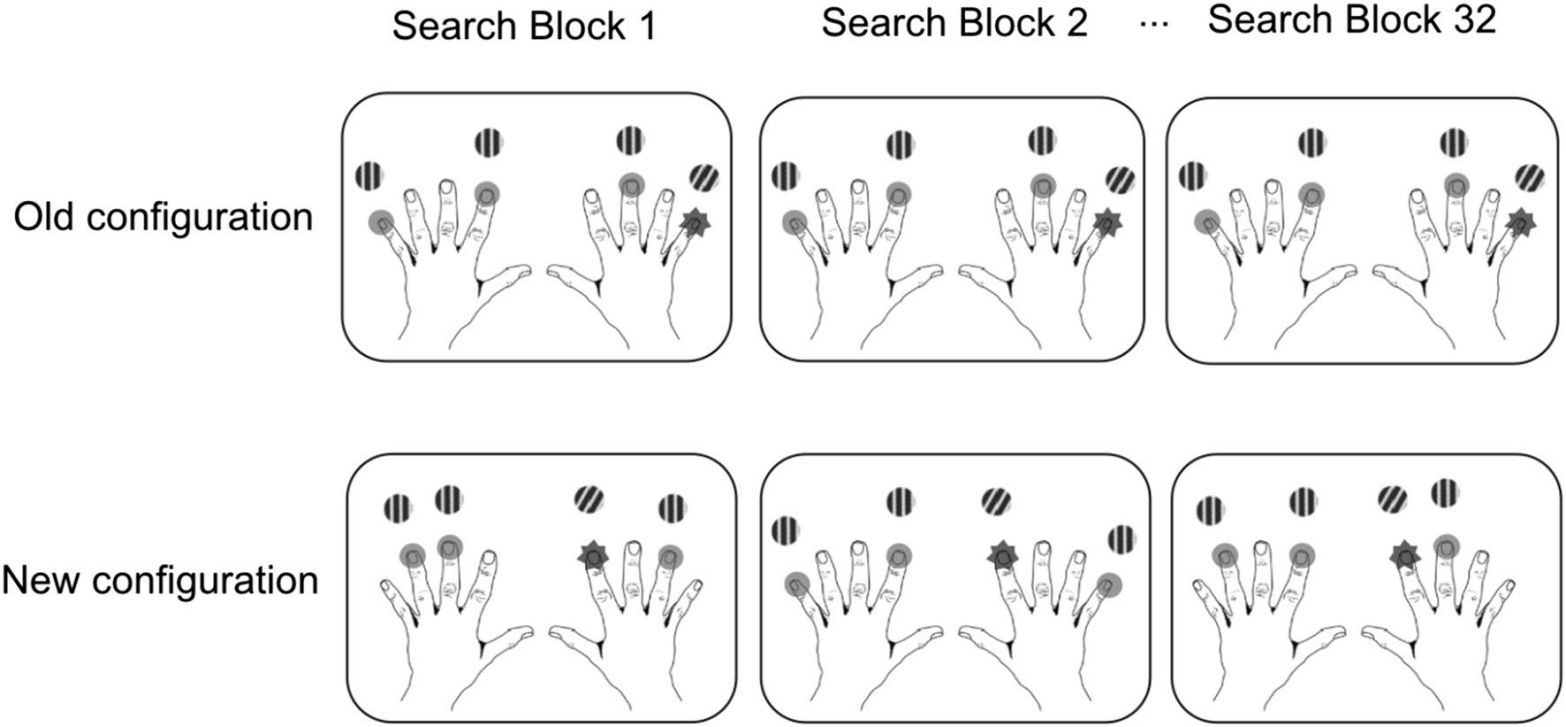
Schematic illustration of the distribution of targets in old and new configurations across search blocks. In old configurations, the target location was constant and paired with constant distractor locations; in new configurations, by contrast, only the target, but not the distractor, locations were held constant across repetitions.

In order to balance stimulus presentations between the left and right hemifields (hands), the search arrays always consisted of two distractors on one side and one target and another distractor on the other side. In total, there were 144 possible configurations to be sampled from. For the repeated contexts (i.e., the old displays), we randomly generated two different sets of four configurations for each participant, one set for the unisensory session (hereafter Set 1) and one for multisensory session (hereafter Set 2). Separate sets of old displays were generated in order to minimize potentially confounding transfer effects across sessions. The four old configurations presented in each block (with eight trials in total) were repeated 32 times across the 32 blocks in each session. For the remaining four new configurations, by contrast, the pairing of the target location with the distractor positions was determined randomly in each block and these configurations changed across blocks. In other words, in old displays (of both sets), the positions of both the target and distractors were fixed throughout the entire session. For new contexts, the positions of the search distractors were randomly generated on each trial anew; that is, these positions bore no predictive information as to the target location, making it impossible for participants to form spatial distractor-target associations. Note, though, that target positions were repeated equally often in new and old configurations (see Fig. 3). That is, in each block of four old and four new trials, four positions, two from each side, were used for targets in the old condition, and the remaining four positions (again two on each side) for new configurations. This was designed to ensure that any performance gains in the old conditions could only be attributed to the effects of repeated spatial arrangements, rather than repeated target positions, in this condition (see, e.g.,^1^, for a similar approach).

### Statistical analysis and modeling of mean RTs

Error trials and trials with extreme RTs (below 200 ms or outside the range of 2.5 standard deviations from individuals’ means) were excluded from RT analyses. Data were averaged across four consecutive blocks into one ‘epoch’ to obtain a reasonably stable estimate of the contextual-cueing effect^1^. Repeated-measures ANOVAs with the factors Trial type (unisensory-visual, multisensory-visual, multisensory-visuotactile), Configuration (old vs. new), and Epoch (1–8) and post-hoc t-tests were used for statistical analysis of mean RTs and exponential model parameters. A Bonferroni correction was applied in case multiple comparisons were conducted.

Given that ANOVAs do not provide a means for identifying the non-linear functions characterizing learning over time, we further fitted the learning curves using a nonlinear, power-law function (see, e.g.,^19^, or more generally the “law of practice” in^30,31^) as follows:

$$RT_{N} = RT_{a} \, + \,(RT_{0} \, - \,RT_{a} ) \, \cdot e^{ - \alpha N} ,$$

where *RT*_*N*_ is the expected value of RT in epoch number *N; RT*_0_ is the response time at the beginning of learning, *RT*_*a*_ is the asymptotic level after learning, and *α* is the learning rate (how fast participants reach their overall learning plateau). Inherently, given all displays are new to participants prior to the experiment, we assume the initial RT at the start of testing (i.e., *RT*_0_) is the same across the various experimental conditions (i.e., old and new configurations for all—unisensory-visual, multisensory-visual, and multisensory-visuotactile—trial types), with *RT*_0_ − *RT*_*a*_ indicating the magnitude of the learning effect (including both general procedural learning of how to perform the task and specific contextual learning).

### Hierarchical drift–diffusion modeling of RT distributions

To understand at which (preattentive, postselective) processing stage(s) contextual cueing operates, we applied drift–diffusion (DD) modeling^32–35^ to individual observers’ RT data. DD models (DDMs) have often been used to infer latent processing stages underlying decision-making. DDMs assume that noisy sensory information is sampled over the time from the environment until the accumulated evidence reaches one of two decision boundaries, triggering a choice decision. The DDM model has multiple parameters: the drift rate parameter *v*, the boundary separation *a*, the initial preference (bias) *z*, and the non-decision time parameter *t*. These parameters reflect different processing stages, which are useful for distinguishing different functional architectures of visual search. (1) In more detail, the drift-rate parameter *v* encapsulates the quality of the evidence driving decision-making: higher drift rates correspond to stronger, more discriminative, evidence entering the decision process, thus yielding both faster responses and greater accuracy. It is important to note that previous contextual-cueing studies adopted mainly difficult search tasks which involve the serial scanning (including sequential eye movements^36,37^) to find the target (typically, search for a uniquely oriented target ‘T’ amongst heterogeneously oriented ‘L’ distractors: a within-form conjunction target). In the present study, by contrast, the target (a Gabor patch tilted by ± 9.2° off the vertical) differed from the homogenous distractors (all vertically oriented Gabors of the same spatial frequency as the target) by a single feature: tilt—thus, rendering this task a relatively easy ‘feature-singleton’ search task^35,38,39^. Previous studies have shown that, for such feature-singleton searches, variations in search speed due inter-trial history (i.e., essentially short-term *memory*) effects, are well captured by the rate parameter in DDMs, irrespective of whether the task requires a simple target-detection (i.e., target-present/absent) response or further processing of the target for its identity to select the response. Consequently, we attribute the drift-rate parameter to the perceptual accumulation of ‘target’ evidence, that is, including both evidence regarding the various items’ status as target versus nontarget (pre-attentive target selection) and evidence as to the target’s response-critical feature (post-selective target identification, e.g., determining the visual target as left- or right-tilted); note that pre-selective (target selection) and post-selective (target identification) processes are notoriously difficult to distinguish in the drift rate parameter^39^. An increased drift rate for old displays would more likely reflect expedited individuation of the singleton target in this condition, rather than facilitated target identification at the subsequent processing stage (which may be assumed to take a relatively fixed amount of time). (2) Evidence accumulation is assumed to start at some point, *z*, situated between two decision boundaries (zero and *a*), where *z* reflects the initial preference for a particular response. Given that the two alternative responses were equally likely in the present study, we assume an unbiased *z* = *a/2.* (3) The boundary separation *a* (i.e., the distance between the two decision boundaries) reflects the amount of information required to trigger a response. Observing a reduced boundary separation (*a*) for old displays would mean that less evidence needed to be accumulated to reach a decision (i.e., the decision criterion is set more liberally), thus essentially expediting responses even if the drift rate was constant. However, given that accuracy in the DDM is determined by the combination of boundary separation and drift rate, a reduced *a* would lead to an increased error rate only in the absence of an effect of old (vs. new) displays on the drift rate. (4) The ‘non-decision’ time parameter *t* refers to the time required for the sensory encoding of the information plus the time necessary for executing the motor response, thus essentially encompassing all components of the response time not directly related to the decision process itself^40^. Assuming that the motor response requirements are similar across experimental conditions, potential differences in non-decision times could be taken to reflect exclusively the initial sensory processing in a visual search task^41^.

By and large, the above predictions concerning the influence of contextual cueing on the DDM rate and boundary-separation parameters are consistent with previous studies that modeled cueing effects in the ‘standard’ (T vs. L’s) visual search paradigm^25,27^, though these studies advocated slightly different interpretations of the drift rate as reflecting pre-attentive processes of target detection^27^ versus post-selective processes of target discrimination^25^. However, given the status of the current search task as feature-singleton or ‘odd-one-out’ search, for which the drift rate has been shown to be the most critical parameter underlying the effects of selection history (independently of whether the task requires simple detection or discrimination of the search target; see references above), finding an effect of contextual cueing on the DDM rate parameter would most likely reflect a beneficial effect on visual target selection.

According to the two alternative accounts sketched in the Introduction, acquired context cues would either reduce the time it takes to attentionally select the target in the visual search display (attentional-guidance account^23^) or reduce the time required to analyze the target and select a response (response-facilitation account^24^). In terms of DDM parameters, a straightforward interpretation of these accounts would be that they predict a contextual-cueing effect on the drift rate *v* (attentional-guidance account) or, respectively, the boundary separation *a* (response-facilitation account).

To gain insight into the process/es that are influenced by contextual cueing, we employed hierarchical drift–diffusion modeling (HDDM), comparing multiple hypothetical models with different underlying mechanisms and examining which model best fitted the behavioral data. The models we evaluated can be divided into four categories:

1. Baseline model: the three key parameters of the DDM – drift rate (*v*), boundary separation (*a*), and the non-decision time (*t*) – were fixed. The other models (2–4) were compared to this baseline to examine for the improvement in RT performance.
2. Partial fixed models: permitted our experimental manipulations of (old vs. new) context and (unisensory vs. multisensory) session/ experience to influence only one or two (of the three) DDM parameters. In this category, we have six models (see Fig. 6).
3. Full varied model: permitted all three DDM parameters (*v, a, t*) to vary across the contexts and sessions.
4. Covariate models: assumed that some parameters change linearly across the learning period (epochs): *par* = *β*_*0*_ + *β*_*1*_(Condition) • Epoch, while the remaining parameters were allowed to vary among conditions, though being invariant across the epochs. Note that coefficient *β*_*1*_ is assumed to be condition-dependent. Here, we estimated seven covariate models separately: three models with one epoch-covariate parameter and two condition-dependent parameters; three models with two epoch-covariate parameters and one condition-dependent parameter; and one model with three epoch-covariate parameters.

We employed the hierarchical DDM toolbox (HDDM) recently developed by Wiecki et al.^42^. The HDDM is a hierarchical Bayesian estimation of drift–diffusion parameters based on the RT distributions of both correct and incorrect responses. Applying Bayesian statistical methods, it simultaneously estimates parameter distributions at both the group level and the individual participant level. We used Markov-chain Monte-Carlo-sampling methods for accurate Bayesian approximation of the posterior distribution of the model parameters (generating 15,000 samples, discarding 5000 samples as burn-in because initial samples are likely to be unreliable due to the selection of a random starting point). We inspected traces of the parameters, their autocorrelation, and computed the R-hat (Gelman-Rubin) convergence statistics to ensure that the models had properly converged^42^. Bayesian hypothesis testing was performed by analyzing the probability mass of the parameter region in question (estimated by the number of samples drawn from the posterior that fall in this region; for example, percentage of posterior samples greater than zero). Bayesian inference was performed on the group mean posteriors^43^.

## Results

### Behavioral measures

**Figure 4:**
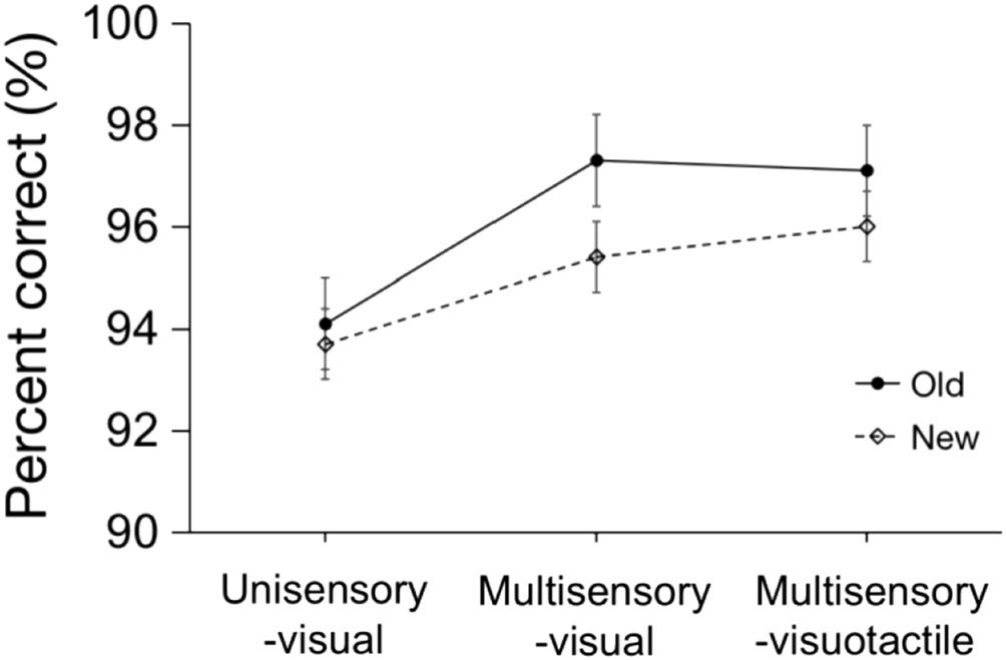
Mean percent correct for old and new configurations on unisensory-visual, multisensory-visual, and multisensory-visuotactile trials. Error bars depict the standard errors of the means (SEM).

*Accuracy*. A repeated-measures ANOVA with the factors Trial type (unisensory-visual, multisensory-visual, multisensory-visuotactile), Configuration (old vs. new), and Epoch (1–8) revealed a main effect of Trial type, *F*(2, 26) = 4.03, *p* = 0.03, *η*_*p*_^2^ = 0.24, with higher accuracy for the multisensory (96.5% ± 0.7% (SEM)) and visual trials (96.3% ± 0.5%) in the multisensory session than the visual trials in the unisensory session (93.9% ± 1%; see Fig. 4). The main effect of Configuration was also significant, *F*(1, 13) = 11.09, *p* = 0.005, *η*_*p*_^2^ = 0.46, with higher accuracy for old versus new configurations (Mean difference = 1.1% ± 0.3%)—thus ruling out potential confounding of the RT cueing effect (see below) by a speed-accuracy trade-off. Neither the main effect of epoch nor any of the interaction effects were significant, all *p*s > 0.29, *η*_*p*_^2^s < 0.09.

**Figure 5:**
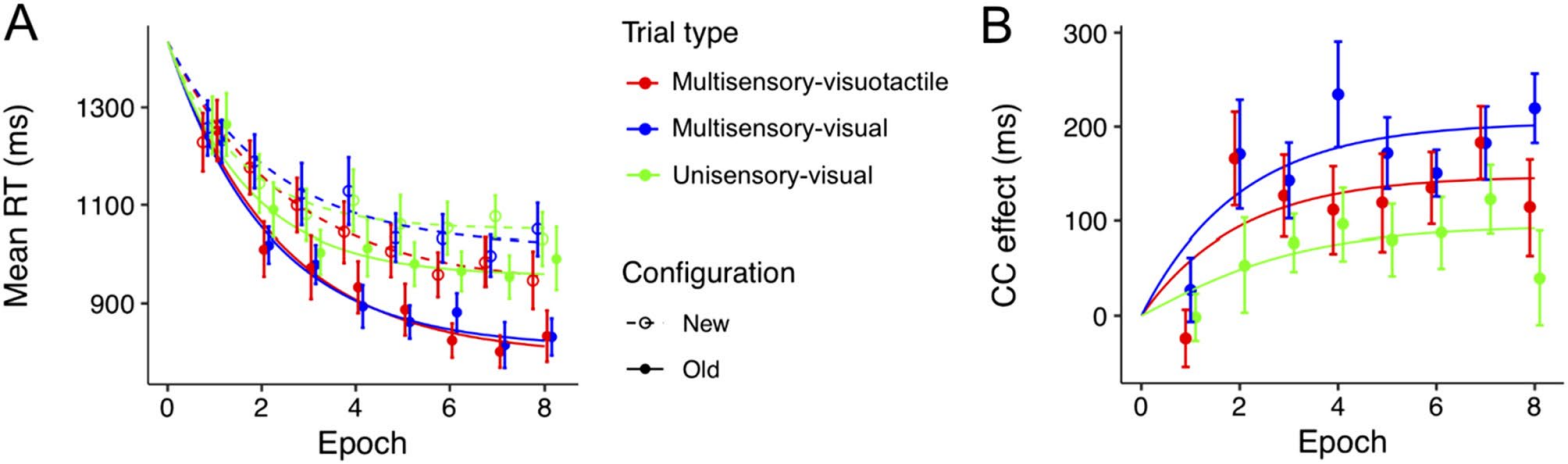
Mean contextual-cueing effects and exponential modeling results. (**A**) Mean RTs for old and new configurations in the three trial-type conditions across epochs (one epoch collapses the RT data across 4 consecutive blocks). The curve represents two-parameter exponential, i.e., power-function fits to the data (see text for explanation). Error bars depict the standard errors of the means *(SEM)*. (**B**) Mean contextual cueing (CC) effects as a function of epoch in the three trial conditions: unisensory-visual, multisensory-visual, and multisensory-visuotactile (green, blue, and red lines, respectively). The subtraction curves ‘old minus new configurations’ for the three trial types (see the corresponding color-coded curves in (**A**) are depicted along with the mean contextual-cueing effects indicated by the individual data points. Note that the fitting of the curves depicted in Fig. 5B was constrained by the (psychological) assumption that there would not be any (acquired) contextual-cueing effect at the beginning of the experiment (i.e., Epoch 0). The fit of model was assessed using the normalized Root Mean Square Error (RMSE), also referred to as the scatter index (SI): SI = (RMSE/average observed value)*100%. The values of SI turned out to be 2.6%, 3.6%, and 4.4% for unisensory-visual, multisensory-visual, and multisensory-visuotactile trials, respectively. Thus, the fit is good for all three conditions, though slightly better for unisensory-visual trials because of the above-mentioned constraint for Epoch 0.

*RT performance.* Extreme RTs occurred in 2.4% of all trials (which were discarded prior to RT analysis). A repeated-measures ANOVA of individuals’ mean RTs with the factors Trial type (unisensory-visual, multisensory-visual, multisensory-visuotactile), Configuration (old vs. new), and Epoch (1–8) revealed significant main effects of Configuration, *F*(1, 13) = 50.10, *p* < 0.001, *η*_*p*_^2^ = 0.79: reflecting shorter RTs to old than to new configurations (mean contextual-cueing effect = 116 ± 16 ms), and of Epoch, *F*(7, 91) = 33.88, *p* < 0.001, *η*_*p*_^2^ = 0.72, indicative of a decrease in RTs as the experiment progressed (*p* < 0.001). The main effect of Trial type was not significant, *F*(2, 26) = 1.64, *p* = 0.21, *η*_*p*_^2^ = 0.11, that is: notwithstanding numerical differences, the overall RTs were largely comparable among the three trial types, unisensory-visual: mean RT = 1068 ± 43 ms, multisensory-visual: mean RT = 1020 ± 35 ms, multisensory-visuotactile: mean RT = 997 ± 39 ms (*t*s < 1.78, *p*s > 0.30, *d*_*z*_s < 0.48). This effectively rules out that, on multisensory-visuotactile trials, participants only relied on the first-presented, tactile stimulus for response. Further, the Configuration by Epoch interaction was significant, *F*(7, 91) = 3.96, *p* < 0.001, *η*_*p*_^2^ = 0.23, with the contextual-cueing effect (the RT difference between old and new configurations) being significant from Epoch 2 onwards (all *p*s < 0.002; in Epoch 1: *p* = 0.99). Importantly, the Trial type by Configuration interaction was significant, *F*(2, 26) = 4.71, *p* = 0.018, *η*_*p*_^2^ = 0.27, due to the contextual-cueing effect being overall more pronounced in the multisensory session (multisensory-visual and multisensory-visuotactile trials: 162 ± 26 ms and 116 ± 20 ms, respectively) than in the unisensory session (69 ± 25 ms, see Fig. 5A). The interaction between Trial type and Epoch and the three-way interaction were not significant, both *p*s > 0.47, *η*_*p*_^2^s < 0.07.

Of note, although the contextual-cueing effect was weaker in unisensory relative to multisensory trials, it was nevertheless reliable (i.e., greater than zero) in the former condition (69-ms effect, one-tailed *t*(13) = 2.78, *p* = 0.008, *d* = 0.74), as were the 162-ms effect in multisensory-visual trials (*t*(13) = 6.21, *p* < 0.001, *d* = 1.66) and the 116-ms effect in multisensory-visuotactile trials (*t*(13) = 5.72, *p* < 0.001, *d* = 1.53). While we thus replicate contextual facilitation of visual search in old displays, importantly, the cueing effect was significantly increased when pure visual displays were presented under multisensory compared to unisensory conditions (162 ± 26 vs. 69 ± 25 ms, *p* = 0.028, *d*_*z*_ = 0.81). In other words, although the displays were identical (namely, purely visual) in the two conditions, the contextual-cueing effect was more than two times larger (on visual trials) when the task involved combined visuo-tactile stimulation in (a random) 50% of trials (see Fig. 5B). Although the contextual-cueing effect for multisensory-visuotactile trials (116 ms) was numerically intermediate between unisensory-visual and multisensory-visual trials, it did not differ significantly from either of these two conditions (116 ± 20 vs. 69 ± 25 ms; *p* = 0.53, *d*_*z*_ = 0.38; 162 ± 26 vs. 116 ± 20 ms; *p* = 0.35, *d*_*z*_ = 0.45).

### Power-law fitting of mean RTs

Next, we compared mean RTs for old and new configurations among the three trial types using an exponential model: *RT*_*N*_ = *RT*_*a*_ + (*RT*_0_ − *RT*_*a*_) • *e*^−*αN*^. Models were computed with three parameters, *RT*_0_, *RT*_*a*_, *α*, and data fitting was obtained for three (trial type: unisensory-visual, multisensory-visual, multisensory-visuotactile) × two (configuration: old, new) conditions (see Fig. 5A). We also computed contextual-cueing effects by subtracting the fitted data in the old- from the data in the new-context condition. These subtractions were performed separately for the three trial types, yielding three (increasing) curves with the final level being higher than the starting level (*RT*_*a*_ > *RT*_0_; see Fig. 5B). These analyses revealed the RT facilitation attributable to contextual learning to be largest for the multisensory-visual trials (200 ms, 95% confidence interval [189, 211), intermediate for multisensory-visuotactile trials (145 ms, CI[132, 158]), and lowest for the unisensory-visual trials (93 ms, CI[84, 102]). This observation was confirmed by a 3 × 2 repeated-measures ANOVA, which yielded a significant Trial type × Configuration interaction, *F*(2, 26) = 3.96, *p* = 0.032, *η*_*p*_^2^ = 0.23. For the *new*-context conditions, post-hoc tests revealed the practice-related improvements in RTs (i.e., parameter *RT*_*0*—_*RT*_*a*_) to be comparable among the three trial types (all *p*s > 0.99, *d*_*z*_s < 0.51). In contrast, for the *old*-context conditions, the practice-related RTs gains were significantly higher for both (old) multisensory-visual versus (old) unisensory-visual trials, *p* = 0.024, *d*_*z*_ = 0.92, and (old) multisensory-visuotactile versus (old) unisensory-visual trials, *p* = 0.04, *d*_*z*_ = 0.87, without a difference between (old) multisensory-visual and (old) multisensory-visuotactile trials, *p* > 0.99, *d*_*z*_ = 0.05.

An analogous repeated-measures ANOVA on the learning rate (only) revealed a main effect of configuration, *F*(1, 13) = 5.28, *p* = 0.039, *η*_*p*_^2^ = 0.29, reflecting a lower, more negative, slope of the function relating RTs to epoch number for old displays (see Fig. 5A). That is, the increase in response speed across epochs was larger for old than for new displays, evidencing ‘true’ contextual learning after removing the contributions of procedural task learning (which would be the same for old and new displays). Of theoretical interest is the absence of a Trial type × Configuration interaction (*p* > 0.59, *η*_*p*_^2^ < 0.04), suggesting that the practice-dependent increase in RT speed attributable to contextual learning is independent of the type of—unisensory versus multisensory—training regime.

In sum, the results from exponential modeling suggest that while multisensory training enhanced the overall magnitude of contextual learning, it did not impact on the speed with which participants acquired the respective, underlying contextual memory representations.

### Hierarchical drift–diffusion modeling

The above analysis of the mean RTs revealed that visual contextual cueing was significantly enhanced by multisensory experience. However, from this analysis alone, it remains unknown at which functional level the enhancement arises. To investigate this, we compared multiple hypothetical HDDM models with different underlying mechanisms and examining which model best fitted the behavioral data. The Deviance Information Criterion (DIC) was used for model comparison. DIC values reflect the best trade-off between the quality of fit and model complexity: models with a higher likelihood and a smaller number of parameters achieve a lower DIC value^43^; a DIC difference of 10 is conventionally considered significant, effectively ruling out the model with the higher value^44,45^.

**Figure 6:**
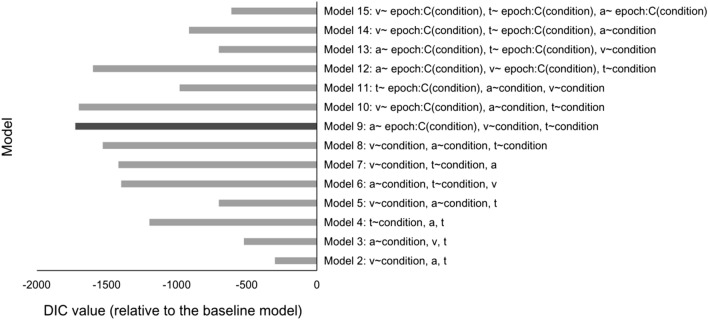
Model comparison. DIC values of several DDM sub-models relative to the baseline DDM model (Model 1, with all parameters fixed; DIC = 7955). v = drift rate; a = boundary separation; t = non-decision time; “ ~ condition” means parameters were dependent on conditions. Here, we have six conditions: 2 configurations (old, new) × 3 trial types (unisensory-visual, multisensory-visual, multisensory-visuotactile). “ ~ epoch:C(condition)” indicates that the parameter is linearly covaried with epoch, while the coefficient β_1_ depends on individual conditions. Model 9 (highlighted by the black bar) exhibited the best fit among all models (DIC = 6230), with a DIC difference of more than 10 relative to Model 10 (with the second lowest DIC = 6257).

**Figure 7:**
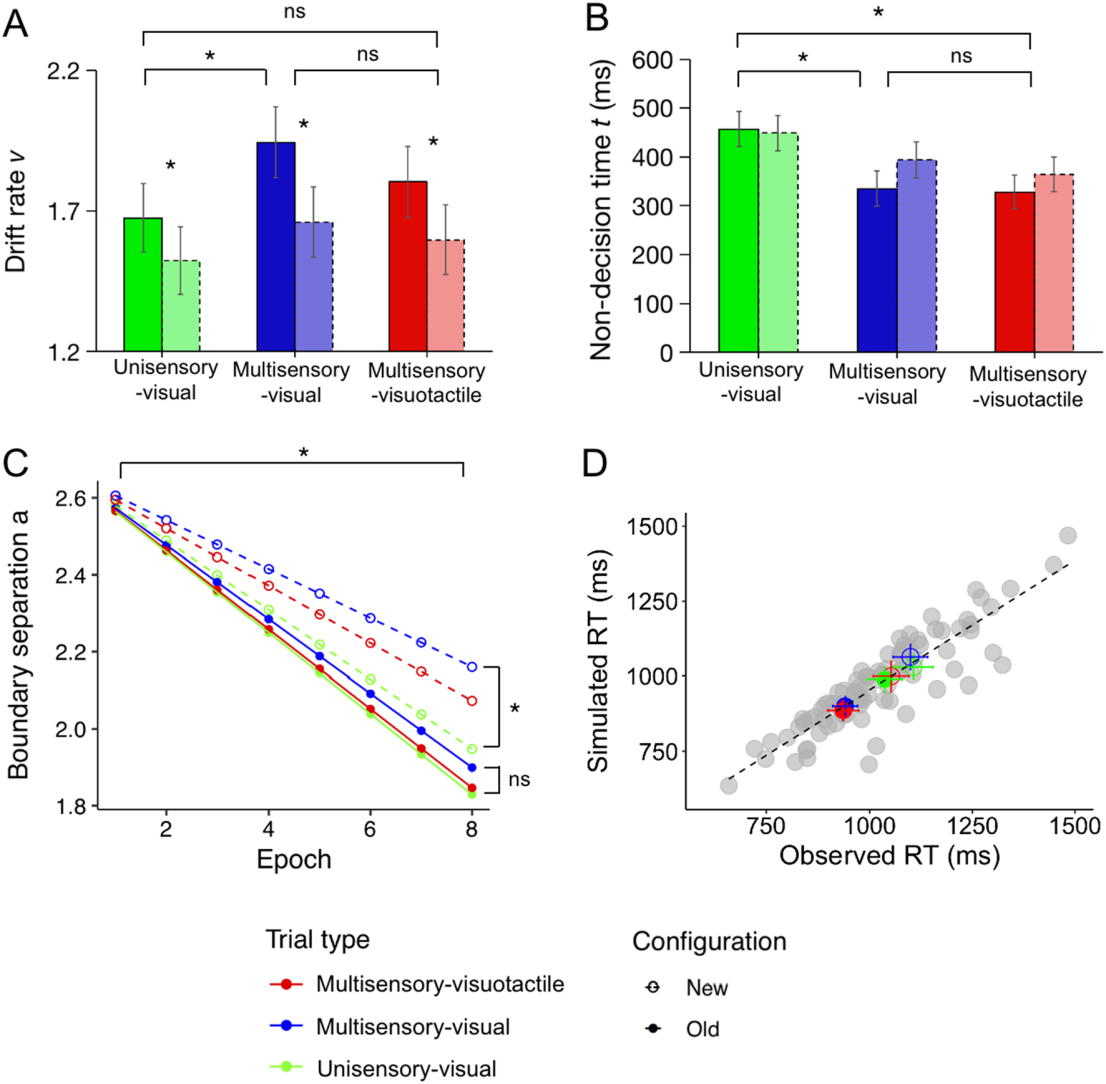
Mean drift rate v (**A**) and non-decision time t (**B**) estimated by hierarchical drift–diffusion modeling as a function of (unisensory-visual, multisensory-visual, multisensory-visuotactile) Trial type and (old, new) Configuration. Error bars depict the standard deviation. Light colored bars indicate new configurations and darker colored bars old configurations. Panel (**C**) shows the function relating boundary separation to epoch number, separately for the experimental (Trial type × Configuration) conditions. Asterisks (*) represent posterior probabilities ≥ 95%. Panel (**D**) depicts predicted (simulated) vs. observed RT data for each observer in all conditions, with model predictions generated from the preferred model. Each observer contributed six data points, one for each (Trial type × Configuration) condition, represented as gray dots. The mean RTs across all observers in each condition are represented by the colored dots. If model predictions match the observed data, the plotted symbols should fall along the diagonal line.

Figure 6 shows the relative DIC values for the 14 DDM sub-models that were compared to the baseline model (Model 1: DIC = 7955). The baseline model assumes that the three DDM parameters (i.e., *v*, *a*, and *t*) are constant across experimental conditions, whereas each sub-model permits one or more parameters to vary across conditions. Note that DIC prioritizes models with fewer parameters, and smaller values represent better model fit. As can be seen, the best model for describing the RT data is the covariate submodel #9 (DIC = 6230), which permitted the decision threshold to vary across epochs in each condition, while the drift rate *v* and the non-decision time *t* were fixed across epochs (though these parameters could vary across the six Trial Type × Configuration conditions). Under this model, the mean drift rates were overall higher for old relative to new displays, as confirmed by Bayesian testing (*P*_*P|D*_ = 98%). Further, there was a systematic effect of the factor Trial Type, with drift rates being lower in unisensory-visual trials relative to multisensory-visual trials (*P*_*P|D*_ = 96%). Importantly, this effect was mainly seen in old configurations (unisensory-visual vs. multisensory-visual: *P*_*P|D*_ = 95%), rather than new configurations (unisensory-visual vs. multisensory-visual: *P*_*P|D*_ = 79%). All other comparisons involving Trial type and Configuration were non-significant, *P*_*P|D*_s < 88% (see also Fig. 7A).

Concerning the non-decision time *t*: While this parameter did not differ between old and new displays (*P*_*P|D*_ = 85%), the non-decision times were reduced in the multisensory relative to the unisensory session (multisensory-visual vs. unisensory-visual trials: *P*_*P|D*_ = 99%; multisensory-visuotactile vs. unisensory-visual trials: *P*_*P|D*_ = 100%). For multisensory-visuotactile trials, a reduction was manifest for both old and new configurations (both *P*_*P|D*_s > 96%)—likely reflecting the fact that the tactile stimulation preceded the onset, and then continued in the presence, of the visual stimulus array. Assuming that the non-decision times reflect initial sensory processing, this result pattern may be attributed to visual processing being enhanced in the presence of tactile information—a kind of crossmodal sensory facilitation effect^46^. In addition to facilitated sensory processing, the previewed tactile items might also have benefitted later processes of response selection, in particular, given that both the tactile and the visual target were associated with identical responses (e.g., both required a left or a right foot-pedal press). Thus, conceivably, the identity of the tactile target might have facilitated visual-target responses, a kind of response-congruency effect. However, what is at odds with this proposal is that there was a reduction of the non-decision times also on multisensory-visual trials (in which no crossmodal response facilitation was possible, because only a single visual target was presented in this condition); and even more importantly, a reduction of non-decision times (in multisensory-visual trials) was seen only for old configurations (*P*_*P|D*_ = 99%), but not new configurations (*P*_*P|D*_ = 86%) (see Fig. 7B). This supports the view advocated above of sensory facilitation from repeated contexts; that is, with repeated visual contexts, the presence of tactile stimulation is not necessary for crossmodal sensory facilitation to occur.

Interestingly, HDDM revealed a significant covariation of epoch with the boundary separation *a*: for all Trial Type × Configuration conditions, there was a significant negative relationship between parameter *a* and epoch number (all *β*s < -0.063, *P*_*P|D*_s = 100%; see Fig. 7C), that is, the boundary separation decreased over the course of the task. In addition, the regression weights were smaller (more negative) for old than for new displays, for all trial types (all *P*_*P|D*_s > 96%). Further, for old configurations, the decrease in boundary separation across task epochs was comparable for all trial types (all *P*_*P|D*_s < 78%), whereas for new configurations the decrease was less pronounced for multisensory-visual than for unisensory-visual trials (*P*_*P|D*_ = 99%). Thus, contextual learning facilitates response selection, over and above the effects arising from non-configural, that is, procedural task learning. This facilitatory effect is independent of whether the underlying context representations were acquired in unisensory or multisensory learning environments. Procedural task learning also facilitates response decisions for new displays—however, apparently less so with multisensory environments, which render responses more cautious with these displays.

Figure 7D plots the predictions deriving from the preferred DDM against the observed RT data. As can be seen, the preferred DDM provided a very close account not only of the group mean RTs, but also of the individual participant RTs in the various (Trial type × Configuration) conditions: all plotted symbols (colored for the group means, gray for individual participants) cluster closely along the diagonal line. Given this, prediction accuracy was not further checked.

## Discussion

The present study investigated the impact of multisensory (versus unisensory) stimulation on the statistical learning of spatial context memories and their effects on visual search performance. We observed a search advantage for targets embedded in old (relative to newly composed) distractor contexts. Strikingly, this search advantage was larger when contextual learning took place in a multisensory environment, suggesting that multisensory experience enhances statistical context memories. Of note, the benefits of multisensory training were evident not only in trials with multisensory, visuo-tactile stimulation; rather, they were even larger on (randomly interleaved) trials with visual stimulation only, suggesting that the visual stimuli alone are sufficient to activate the corresponding multisensory context representations and make them available to the ongoing search. Our (‘exponential’) modeling approach showed that multisensory training particularly increased the magnitude of the cueing effect. In other words, multisensory experience leads to the acquisition of stronger context memories (contextual associations) than are obtained with unisensory stimulation alone! These results significantly extend our understanding of contextual cueing (e.g.,^1,3,6,7,23^): we demonstrate, for the first time, that visual search in predictive (relative to random) target-distractor arrangements is particularly enhanced when observers have multisensory (relative to unisensory) experience, showing that (enhanced) search-guiding visuo-spatial representations may be formed based on inputs from different—visual and tactile—modalities.

In order to investigate at what functional level(s)—search guidance and/or response facilitation—such memory representations interact with visual task performance, we drew on a new, hierarchical drift–diffusion modeling (HDDM) framework to examine how well a variety DDM models could account for the observed RT data. In general agreement with prior DDM studies of contextual cueing in the standard (T vs. L’s) paradigm^25,27^, we found that the acquisition of context cues affects both the rate at which decision-relevant evidence is accumulated—the rate is increased for old (i.e., learnt) vs. new configurations—and the boundary separation, which, over time on task, reduces more markedly for old than for new configurations (i.e., it shows a configural over and above a procedural learning effect), while having little effect on the non-decision times. The boundary-separation effect would support a decision-threshold, or response-facilitation, account of the contextual cuing effect^24^. And the drift rate effect we (and the previous studies) observed is consistent with the idea that contextual memory increases the speed at which target information is accumulated, which we take to mean that contextual cueing supports the guidance of attention to the response-critical item^1,47,48^. Looked at the results on an individual participant basis (see Supplementary Table S1), for eight (of the total of 14) observers, the cueing effects were equally well explained by models that incorporated either a difference in boundary separation only or one in drift rate between old and new configurations only; and for each three observers, the effects were better accounted for by a model that incorporated varied drift rates or, respectively, varied boundary separations. Our results thus provide equal support to both the attentional-guidance and response-facilitation accounts of the contextual-cueing effect.

While this is generally in line with previous work, importantly, we found strong evidence in favor of the idea that multisensory training specifically facilitates the attentional-guidance effect. In more detail, we found that the critical stage that determined the observed pattern of multisensory facilitation was related to the process of evidence accumulation (as well as non-decision time), with old configurations in multisensory-visual trials expediting the rate of evidence accumulation over and above that with unisensory-visual trials (where both types of trial present only the same, visual stimuli). Based on their findings on pop-out visual search, Geyer et al.^23^ suggested that learnt contexts top-down boost the ‘salience’ of the target item, thus expediting attentional selection of and, thus, focal-attentional extraction of response-relevant information from this item. Of note, salience signals, which drive attentional selection, are considered to be ‘feature-blind’, that is, the features characterizing a selected item become available only in a further, focal-attentional analysis stage. Applying this notion to the current multisensory paradigm, the finding of an increased drift rate (and reduced non-decision times) by old configurations as a result of multisensory (as compared to unisensory) training would mean that *visual* context memories acquired through multisensory (i.e., combined visual and tactile) experience provide a greater top-down boost to target ‘salience’, thus expediting the guidance of focal attention to the target. In other words, *visual* context memories acquired through multisensory experience render a more robust cueing effect than memories acquired through unisensory experience, for instance, because (i) multisensory experience leads to stronger or more readily retrievable memory representations of individual distractor-target configurations, and/or (ii) the learning of a greater number of old configurations. Evidence for both effects of multisensory learning was provided by a comparison, between the unisensory and multisensory conditions, of the effective number of old configurations that gave rise to a cueing effect and of the size of the cueing effect the effective old configurations generated. This analysis (see “Single-display analysis” in Supplementary Information) revealed the number of effective old configurations to be substantially (some 25%) higher in the multisensory-visual versus the unisensory-visual condition (see Fig. S1-A). Additionally, the median contextual-cueing effect per effectively learnt display was (25%) greater following multisensory as compared to unisensory learning, indicative of multisensory learning leading not only to a greater number but also to more effective context-memory representations (see Fig. S1-B).

Of note, while multisensory learning experience increased the rate of evidence accumulation from purely visual displays, it did not lead to a reduced boundary separation compared to unisensory learning if anything, there was a differential boundary-separation effect only for new configurations, where the reduction in separation was however less, rather than more, marked with multisensory learning, indicative of more cautious, rather than more liberal, decision making). Given this, we propose that multisensory learning experience exclusively influences (pre-selective) attentional guidance, rather (post-selective) response selection.

There have been multiple demonstrations that multisensory experience can subsequently facilitate unisensory processing, albeit using different methodological approaches and testing different (auditory, visual) modalities (for review, see, e.g.,^49^). For example, exposure to audio-visual stimuli can change the way auditory or visual stimuli are processed subsequently even in isolation, indicative of continuous modification of unisensory representations by multisensory relationships^50,51^. Similarly, we found that (purely) visual search was facilitated when the presentation of both predictive tactile and predictive visual distractor-target configurations was randomly intermixed with the presentation of purely visual configurations. Given the tactile configuration preceded the visual configuration (on multisensory-visuotactile trials) by 450 ms, permitting the tactile stimuli to be (optimally) re-mapped from the initially somatotopically sensed format into external coordinate frame shared with the visual items^11,13^, it is likely that the locations of the visual and tactile stimuli (on visuotactile trials) were co-located in a common, supra-modal representation that mediates cross-modal contextual cueing. The provision of exactly the same spatial information by multisensory sources did not only increase the number of effectively learnt distractor-target patterns, but it also led to a strengthened, or better consolidated, representation of each learnt pattern in visual long-term memory, thereby boosting attentional guidance even when the memory representation is activated by the presentation of a purely visual display (on multisensory-visual trials).

The latter is also supported by evidence from the neural level. It is well established that visuo-spatial memories are strongly dependent on the integrity of the hippocampus in rats and humans^52–55^ and that contextual cueing in humans is especially impaired after damage of the medial temporal lobes (MTL)^56,57^. Recent findings indicate that the hippocampus is also involved in signaling cross-modal predictions, which in turn influence sensory systems^58,59^. For instance, the hippocampus may learn multisensory associations which may then send feedback signals to visual areas^60–63^, thereby leading to the cortical reinstatement of unimodally predicted target-distractor associations. Alternatively, predictive signals generated across sensory systems may optimize the functional connectivity between specialized cortical sensory modules. For instance, sensory redundant stimulus pairs, such as voices and faces, have been shown to induce specific multisensory associations resulting in increased functional coupling between the voice and face areas, and these associative representations subsequently became available for unisensory (voice or face) recognition^50^. Thus, the enhancement of visual contextual cueing following multisensory learning experience, as observed in the present study, might reflect more robust, stronger representations of context memory in the visual sensory regions.

In summary, the present study shows that multisensory, visuo-tactile training facilitates the processing (detection and perceptual analysis) of a visual target embedded in a repeated context of visual distractor items, even when the visual arrays are presented in isolation, without accompanying tactile stimulation. Importantly, multisensory training gives rise to contextual-cueing effects for purely visual stimuli that surpass the facilitation deriving from training under unisensory visual conditions, both in terms of the number of effectively learnt distractor-target configurations and in terms of the size of the cueing effect each learnt configuration generates. Drift–diffusion modeling suggested that multisensory (relative to unisensory) training enhances contextual cueing primarily by facilitating the attentional-guidance stage of the search process, as evidenced by a higher rate of evidence accumulation towards the required decision. (By contrast, facilitation of response selection appears to play an equal role in all, uni- and multisensory, training conditions, as evidenced by a comparable reduction in the separation of the decision boundaries in the repeated trials.) Multisensory training appears to enhance contextual cueing by two related mechanisms: being presented with convergent (co-located) spatial patterns simultaneously in multiple (the tactile and visual) modalities increases the number of repeated distractor-target layouts that are effectively learnt; at the same time, it also strengthens the representations of learnt layouts in visual long-term memory. As a result, these representations can subsequently be more reliably triggered by the presentation of purely visual patterns, thus top-down boosting attentional guidance towards the target location.

## Supplementary Information

Supplementary Information

## Data Availability

The data supporting the findings of the study and the statistical analysis code used in the manuscript are available in the Open Science Framework repository at https://osf.io/hmnp9/.
