# Supplementary material for: Multisensory visuo-tactile context learning enhances the guidance of unisensory visual search
Source: Sci Rep. 2021 May 3;11:9439. doi: 10.1038/s41598-021-88946-6 (PMC8093296; doi:10.1038/s41598-021-88946-6)
Supplement: Supplementary file 1 — Supplementary Information [file 41598_2021_88946_MOESM1_ESM.docx]

Supplementary Information

**Multisensory visuo-tactile context learning enhances the guidance of unisensory visual search**

Siyi Chen, Zhuanghua Shi, Hermann J. Müller, & Thomas Geyer

Ludwig-Maximilians-Universität München, 80802 München, Germany

Running title: Visuotactile context learning enhances visual contextual learning

**DDM model comparison**

To evaluate which parameter (drift rate vs. boundary separation) mainly drives the contextual cueing effect, based on the best model #9 reported above we compared the fits of two versions of our models: one that changed boundary separation (*a*) as fixed across old and new configurations but with drift rate (*v*) varied across configurations, another that changed drift rate (*v*) as fixed for the (old and new) configurations but with boundary separation (*a*) varied across configurations. Other aspects of the two models were kept the same (also permitting the boundary separation to vary across epochs in each trial type while the non-decision time *t* was allowed to vary across the six Trial Type × Configuration conditions). By comparing the fits of the two versions of the diffusion model to the individual observer data, we were able to identify important common principles underlying the contextual cuing effect. As can be seen from **Supplementary Table S1**, for eight (of the total of 14) observers, the cueing effects were equally well explained by models that incorporated either a difference in boundary separation or one in drift rate between old and new configurations; and for each three observers, the effects were better accounted for by a model that incorporated varied drift rates or, respectively, varied boundary separations. This would suggest that (in the majority of 60% of observers) contextual cueing is driven equally by changes in both parameters.

**Supplementary Table S1.** DIC values associated with each model for each observer, with either drift rate (*v*) or boundary separation (*a*) allowed to vary across old and new displays in each model.

| Observer | Parameters varying between old and new displays | | DIC difference (*v*>*a*) |
| --- | --- | --- | --- |
|  | *v* | *a* |  |
| S1 | 312 | 309 | 3 |
| S2 | 515 | 511 | 4 |
| S3 | 248 | 247 | 1 |
| S4 | 797 | 792 | 5 |
| S5 | -5 | 22 | -27 |
| S6 | 547 | 549 | -2 |
| S7 | 799 | 793 | 6 |
| S8 | 147 | 160 | -13 |
| S9 | 780 | 783 | -3 |
| S10 | 696 | 688 | 8 |
| S11 | 454 | 445 | 9 |
| S12 | 406 | 402 | 4 |
| S13 | 205 | 216 | -11 |
| S14 | 289 | 286 | 3 |

Note. Models are identified in terms of the DDM parameters that were allowed to vary between old and new displays. Smaller DIC values are indicative of better model fit. Differences in DIC values greater than 10 definitely rule out the model with the higher value; differences between 5 and 10 are substantial; and differences less than 5 are indicative of the models being virtually indistinguishable in terms of overall fit ^1^. Applying these criteria (as can be seen from the differential DIC values in the right-hand column), for eight (of the total of 14) observers (S1, S2, S3, S4, S6, S9, S12, S14), the cueing effects were equally well explained by models that incorporated either a difference in boundary separation or one in drift rate between old and new displays; and for each three observers, the effects were better accounted for by a model that incorporated varied drift rates (S5, S8, S13) or, respectively, varied boundary separations (S7, S10, S11).

**Single-display analysis**

There are two possible ways in which multisensory learning may be expected to enhance visual contextual cueing: it may increase the number of repeated distractor-target configurations that are effectively represented in context memory and thus produce a cueing effect; and/or for each such representation, it my increase the effectiveness of the cueing it brings about when activated by the presentation of the respective visual configuration. To test for these two possibilities, we conducted analyses at the single-display level to determine, individually for each participant, the number of repeated configurations that generated a contextual-cueing effect greater than zero (i.e., ‘effective’ learnt old configurations) and assessed the magnitude of the cueing-effect per effectively learnt configuration. A given repeated configuration was classified as generating effective cueing if a participant’s mean RT for this configuration was less than her/his mean RT across all non-repeated configurations. In addition, we determined the magnitude of contextual cueing per effective configuration by calculating the difference in RTs between effective repeated and non-repeated (i.e., by definition ‘non-effective’) configurations. The results are depicted in the **Supplementary** **Fig. S1**.


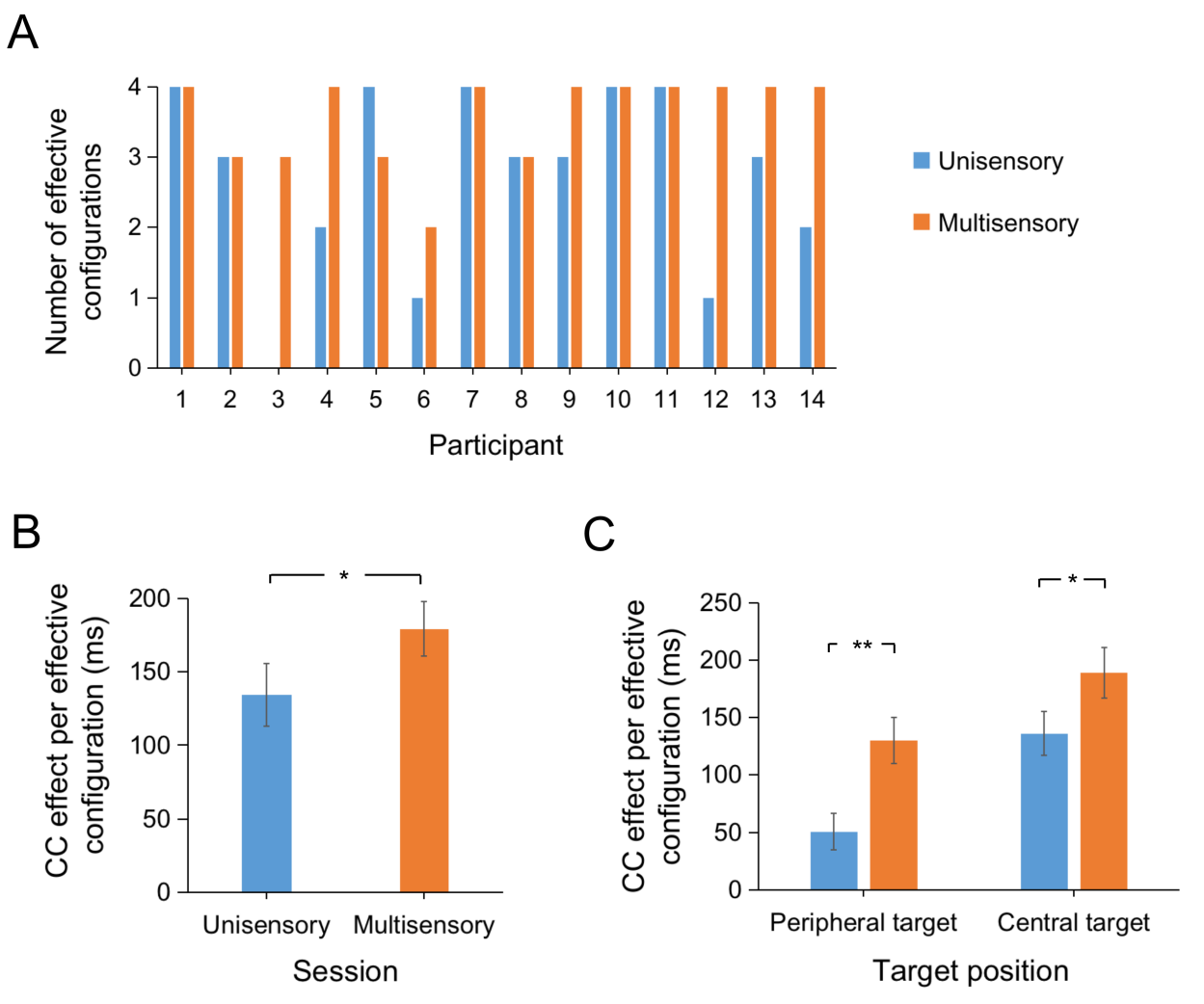


**Supplementary Fig. S1** *Results from single-display analysis.* ***A:*** *Number of repeated displays that generated a RT contextual-cueing effect (RTs non-repeated display minus RT repeated display) in the presence of (purely) visual items separately for the unisensory and multisensory sessions.* ***B:*** *Median contextual-cueing (CC) effects per ‘effective’ repeated configuration (giving rise to a positive CC effect relative to the baseline of non-repeated displays) in the uni- and multisensory sessions.* ***C:*** *Mean CC effect for peripheral (outer) vs. central (inner) targets in the uni- and multisensory conditions. Error bars depict the standard errors of the means. Asterisks represent significance levels of p < .05 (*) and p < .001 (**).*

The results revealed the number of effective repeated configurations to increase significantly from 2.71 ± 0.35 (SEM) in the unisensory session to 3.57 ± 0.17 in the multisensory session, one-tailed *t* (13) = -2.60, *p* = .01, *d* = -0.70. This finding is illustrated in **Fig. S1-A**, which shows that distributions of effective contextual-cueing displays between the unisensory and multisensory sessions are clearly different: seven out of the 14 observers acquired a higher number of effective configurations in the multisensory vs. the unisensory session, with six participants learning the same number of (repeated) display configuration in the two sessions and one participant learning a higher number of effective configurations in the unisensory session. This difference was even more pronounced when we used a more stringent criterion for establishing what an ‘effective display’ is, that is, when a repeated configuration was classified as generating effective cueing only if RTs for this display fell below the 99% confidence interval (CI) of the individual participant’s mean RT for non-repeated configurations (for this approach, see, e.g., ^2-3^). Using this criterion, the difference in the number of effective repeated configurations amounted to 2.93 ± 0.25 in the multisensory session vs. 1.64 ± 0.25 configurations in the unisensory session: *t* (13) = 4.84, *p* < .001, *d* = -1.29; 12 out of the 14 observers acquired a higher number of effective configurations in the multisensory vs. the unisensory session, with two participants learning the same number in the two sessions.

In terms of the (median) magnitude of the contextual-cueing effect (i.e., RT(non-repeated) minus RT(repeated) for the effectively learnt configurations (as defined above), participants displayed a larger cueing effect for purely visual displays in the multisensory than in the unisensory session (see **Fig. S1-B**; unisensory session: 134 ± 22 ms; multisensory session: 179 ± 19 ms), one-tailed *t* (13) = 1.95, *p* = .037, *d* = 0.52. The median magnitudes (calculated per participant) were compared because of the small number of effective configurations (2.71 vs. 3.57 on average) across which the size of the cueing effect was calculated. [Note, though, that the magnitude of the cueing effect per effective display identified by the more stringent criterion (see above) did no longer differ between the two (uni- and multisensory) learning conditions (*p* = .46, *d* = -0.03). However, the absence of a difference is unsurprising given the analysis focused exclusively on those displays (in the distribution of displays with a cueing effect > 0) that produced near-optimal cueing.] Thus, the single-display analysis shows that both the number of effectively learnt configurations as well as the magnitude of the cueing effect that each one generates contribute to the enhanced learning effect seen with multisensory as compared to unisensory training.

More detailed, target-position-dependent analyses suggested that the gains in contextual cueing (per effective display) from multisensory training were more marked for outer (the left- or right-most) than for inner target positions (see **Fig. S1-C**), which also showed a reduced cueing effect overall (consistent with Geyer et al. ^4^). Besides a main effect of Learning session (*F*(1, 13) = 12.28, *p* = .003, *η_p_^2^* = .49) reflecting a larger cueing effect following multisensory versus unisensory learning (mean difference of 66 ± 19 ms). A two-way ANOVA revealed the main effect of (inner, outer) Target position to be significant, *F*(1, 13) = 35.84, *p* < .001, *η_p_^2^* = .73. Although the difference between the unisensory and multisensory sessions was statistically more robust for configurations with an outer (79 ms; *t* (13) = -3.96, *p* < .001, *d* = -1.06) versus an inner target position (53 ms; *t* (13) = -1.79, *p* = 0.048, *d* = -0.48), the Session × Position interaction turned out non-significant, *F*(1, 13) = 0.62, *p* = .45, *η_p_^2^* = .05.

**References**

1 Spiegelhalter, D. J., Best, N. G., Carlin, B. P., & van der Linde, A. Bayesian measures of model complexity and fit. J*ournal of the Royal Statistical Society: Series B (Statistical Methodology)* **64**, 583–639(2002).

2 Smyth, A. C., & Shanks, D. R. Awareness in contextual cuing with extended and concurrent explicit tests. *Memory & Cognition* **36**, 403–415(2008).

3 Geyer, T., Müller, H. J., Assumpcao, L., & Gais, S. Sleep-effects on implicit and explicit memory in repeated visual search. *PloS One* **8**, e69953(2013).

4 Geyer, T., Rostami, P., Sogerer, L., Schlagbauer, B., & Müller, H. J. Task-based memory systems in contextual-cueing of visual search and explicit recognition. *Sci Rep* **10**, 16527 (2020).
